# Effect of intravenous induction with different doses of Esketamine combined with propofol and sufentanil on intraocular pressure among pediatric strabismus surgery: a randomized clinical trial

**DOI:** 10.1186/s12871-023-02238-2

**Published:** 2023-08-15

**Authors:** Jun Luo, Kuoqi Yin, Dinghuan Zhao, Zhao Zhang, Ruiqiang Sun

**Affiliations:** Department of Anesthesiology, Tianjin Eye Hospital, 4 Gansu Road, 300020 Tianjin, China

**Keywords:** Intraocular pressure, Esketamine, Propofol, Anesthesia

## Abstract

**Background:**

It is well-established that maintaining stable intraocular pressure (IOP) within the normal range during ophthalmic surgery is important. Esketamine is a commonly used drug in pediatric general anesthesia due to its good analgesic and sedative effects. However, its application in ophthalmic surgery is limited because it can increase IOP. The effect of esketamine combined with other common anesthetics on IOP has been underinvestigated. This study aimed to investigate the effect of different doses of esketamine combined with propofol and sufentanil on IOP during intravenous induction of general anesthesia for pediatric strabismus surgery.

**Methods:**

A total of 181 children with strabismus undergoing unilateral eye surgery under general anesthesia were recruited. Intravenous induction included the use of sufentanil 0.1 µg/kg, propofol 3 mg/kg, and esketamine. Base on the dosage of esketamine, the patients were randomly allocated into three groups: esketamine low (EL) group with 0.25 mg/kg (n = 62), esketamine high (EH) group with 0.5 mg/kg (n = 60), and normal saline (NS) group (n = 59). Hemodynamic parameters, respiratory parameters, and IOP of the non-surgical eye were recorded and compared among the three groups at different time points: before induction (T_0_), 1 min after induction but before laryngeal mask insertion (T_1_), immediately after laryngeal mask insertion (T_2_), and 2 min after laryngeal mask insertion (T_3_).

**Results:**

There were no significant differences in age, gender, body mass index (BMI), and respiratory parameters among the three groups at T_0_. The IOP at T_1_, T_2_, and T_3_ was lower than that at T_0_ in all three groups. The EH group (12.6 ± 1.6 mmHg) had a significantly higher IOP than the EL group (12.0 ± 1.6 mmHg) and the NS group (11.6 ± 1.7 mmHg) at T_1_. However, no difference was found between the EL and NS groups at any time point. Systolic blood pressure (SBP) and heart rate (HR) at T_1_, T_2_, and T_3_ were lower than at baseline, and SBP and HR were higher at T_2_ than at T_1_. Additionally, the EH group had a significantly higher HR at T_1_ than the other two groups. There was no significant difference in diastolic blood pressure (DBP) among the three groups at any time point.

**Conclusion:**

Propofol combined with sufentanil significantly decreased IOP during the induction of general anesthesia. Although a dose of 0.5 mg/kg esketamine elevated IOP compared to the low-dose and control groups after induction, the IOP remained lower than baseline. 0.25 mg/kg esketamine combined with propofol and sufentanil had little effect on IOP. Therefore, we advocate that a maximum dose of 0.5 mg/kg esketamine combined with propofol and sufentanil will not elevate IOP compared to baseline in pediatric strabismus surgery.

**Trial registration:**

The registration number is ChiCTR2200066586 at Chictr.org.cn. Registry on 09/12/2022.

## Introduction

Stable intraocular pressure is beneficial for maintaining the shape and function of the eyeball, essential for the prognosis of patients undergoing ophthalmic surgery or those with ophthalmic diseases. The sudden increase in intraocular pressure during the induction period can lead to nausea, vomiting, central retinal artery occlusion, and even prolapse of intraocular contents. Conversely, excessively low intraocular pressure can induce complications such as retinal detachment and choroidal edema [1]. During general anesthesia, especially during the induction period, anesthesiologists should maintain circulatory and respiratory stability and understand and pay attention to the changes in intraocular pressure while minimizing fluctuations.

Strabismus surgery in children is often performed under general anesthesia, and the choice of induction medication can have different impacts on intraocular pressure [2]. Among the commonly used induction drugs, propofol and opioids have been shown to reduce intraocular pressure [3], while ketamine has the effect of increasing IOP. As a novel dextro-isomer of ketamine, esketamine has stronger anesthetic and analgesic effects and a lower impact on the cardiovascular, respiratory, and neurological systems [4, 5], making it more suitable for pediatric general anesthesia than ketamine [6]. The mechanism of esketamine in increasing intraocular pressure is similar to that of ketamine, as it activates the sympathetic nerve. Current research on the regulatory effect of ketamine on intraocular pressure remains controversial. Consequently, no consensus has been reached regarding whether esketamine can raise intraocular pressure, which limits its application in pediatric ophthalmic anesthesia.

In clinical practice, esketamine is often used in combination with other anesthetic drugs (e.g., opioids, propofol, etc.) rather than being used alone [7, 8]. Therefore, we hypothesized that the combined use of esketamine, propofol, and sufentanil would not elevate intraocular pressure during induction. This study aimed to compare the effects of different doses of esketamine combined with propofol and sufentanil on intraocular pressure during intravenous induction of general anesthesia for pediatric strabismus surgery. Additionally, we aimed to investigate the optimal dose of esketamine for pediatric ophthalmic surgery and provide references and a basis for further clinical research and application.

## Materials and methods

### Study population

The institutional review board of Tianjin Eye Hospital approved this prospective, randomized, double-blind study, registered in the Chinese Clinical Trial Registry on 09/12/2022 (ChiCTR2200066586). Guardians of all participants signed informed consent after obtaining a detailed understanding of the study objectives, procedures, and potential risks.

**Fig. 1:**
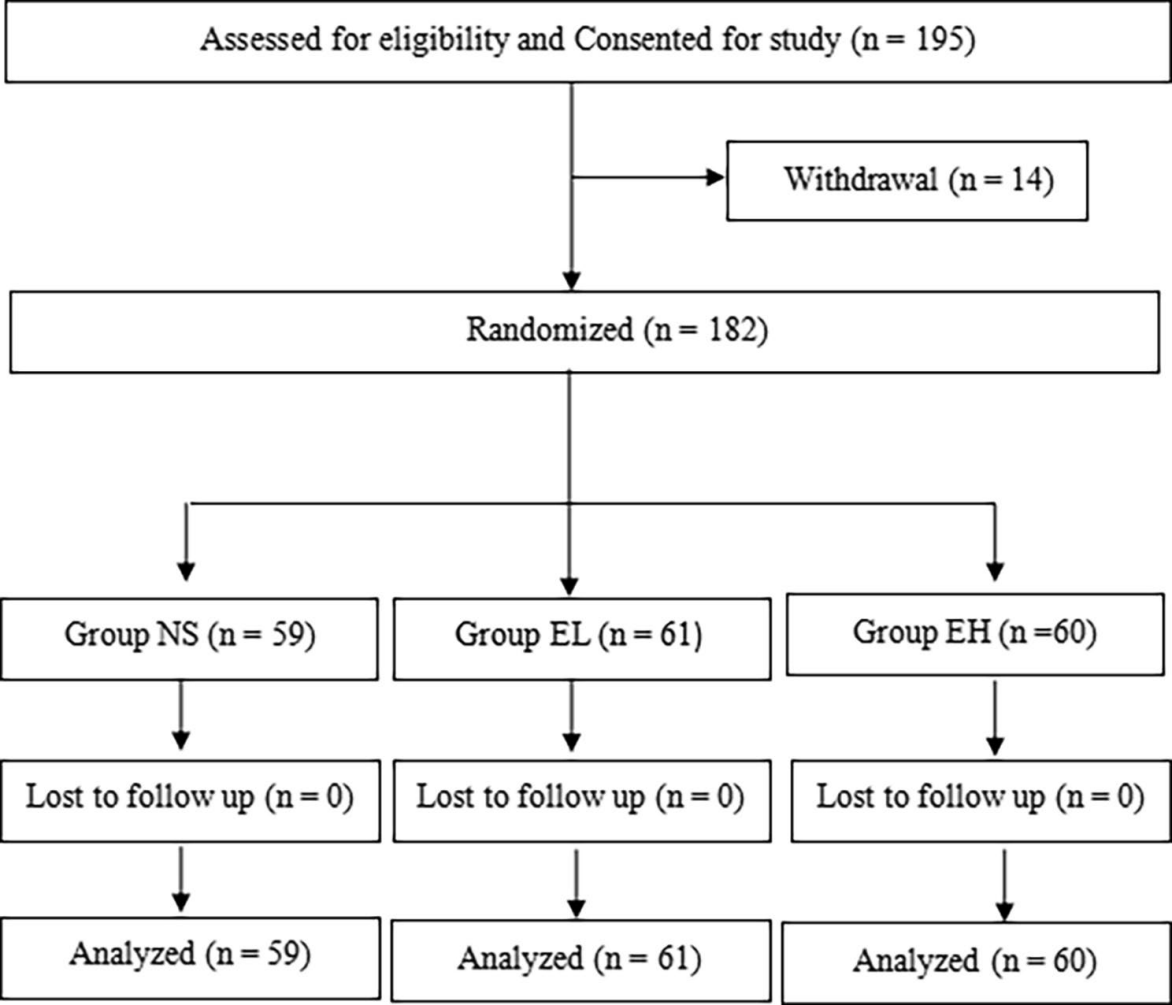
CONSORT diagram showing the flow of participant recruitment throughout each stage of the randomized trial. Patients in EL group was injected with esketamine 0.25 mg/kg, EH group was injected with esketamine 0.5 mg/kg, and NS group was injected with normal saline

A total of 181 children with strabismus undergoing monocular surgery under general anesthesia were recruited at our hospital from December 2022 to February 2023. The inclusion criteria were as follows: (1) aged 7 to 12 years; (2) grade I or II in the American Society of Anesthesiologists (ASA) physical status classification. Patients were excluded if they (1) had a history of glaucoma, keratopathy, retinopathy, neuromuscular disease, difficult airway, or upper respiratory tract infection within 2 weeks before surgery; (2) were allergic to related general anesthesia or local anesthesia drugs, or had received medication affecting IOP within 2 days before surgery; (3) were unable to cooperate with IOP measurement under topical anesthesia before the induction of general anesthesia. More details are provided in Fig. 1.

### Study methods

Using a random number table created by statisticians using SPSS statistical software, 181 patients were allocated into three groups: esketamine low (EL) group, esketamine high (EH) group, and normal saline (NS) group. All patients had routine fasting for 8 h and liquid fasting for 2 h before surgery. Penehyclidine hydrochloride 0.01 mg/kg was injected intramuscularly 30 min before anesthesia. After admission to the operating room, venous access was established, and noninvasive blood pressure (BP), electrocardiogram (ECG), and pulse oxygen saturation (SpO_2_) were monitored. General anesthesia induction began after 3 min of oxygen inhalation (oxygen flow 6 L/min, FiO_2_ = 100%). An anesthesiologist blinded to the group allocation performed the induction, administering an intravenous injection of propofol 3 mg/kg and sufentanil 0.1 µg/kg. A laboratory staff member prepared the induction drugs (uniformly labeled and with the same volume of 2ml) and handed them over to the anesthesiologist. After the loss of consciousness and eyelash reflex, the EL group received an injection of esketamine 0.25 mg/kg, the EH group received an injection of esketamine 0.5 mg/kg, and the NS group received an injection of normal saline. During the induction, the airway was kept patent, we used assisted ventilation to maintain end-tidal carbon dioxide pressure (PETCO_2_) at 35-45mmHg when necessary. A laryngeal mask airway (LMA) was implanted if there was no body movement with jaw-lift after 1 min of administration. If necessary, 0.5 mg/kg propofol could be added, and the anesthesia machine was connected after successful LMA implantation. Combined intravenous-inhalation anesthesia was maintained during surgery with 1-2% sevoflurane and 50–100 µg/kg/min propofol. Spontaneous breathing was preserved, and the depth of anesthesia was adjusted according to the vital signs.

### Data collection

Preoperative general indicators included age, gender, weight, body mass index (BMI), and ASA classification. Systolic blood pressure (SBP), diastolic blood pressure (DBP), heart rate (HR), tidal volume (VT), PETCO_2_, and non-surgery eye IOP were recorded before induction (T_0_), 1 min after induction but before laryngeal mask insertion (T_1_), immediately after laryngeal mask insertion (T_2_) and 2 min after laryngeal mask insertion (T_3_).

The primary outcome of this study is IOP. IOP at each time point was measured by a skilled ophthalmologist blinded to the group allocation. Before the induction of anesthesia, the patient’s eye was topically anesthetized with 2 drops of 2% lidocaine. The IOP was measured three times by Tono-Pen AVIA intraocular pressure meter (Tono-Pen AVIA, Reichert Technologies, Depew, NY, USA), and the mean values were taken.

### Statistical analysis

Based on the results from a pilot study, the PASS software was used for sample size calculation. With α = 0.05 and β = 0.2, it was determined that 54 cases in each group were needed. Considering a 20% potential loss to follow-up, a total of 195 patients were required for this study.

The normal distribution was assessed using a quantile-quantile plot (Q-Q plot) and the Kolmogorov-Smirnov test. Continuous variables were presented as mean ± standard deviation (SD) for normally distributed data or median and interquartile range (IQR) for non-normally distributed data. Numerical differences among the three groups were analyzed using ANOVA for normally distributed variables with homogeneity of variance. Analysis of variance was used for inter-group comparisons at the same time point, and Bonferroni correction was applied for intra-group comparisons at different time points. The chi-square test was used for analyzing categorical data. A p-value less than 0.05 was statistically significant. All statistical analyses were performed using SPSS, Version 25.0 (SPSS Inc., Chicago, IL, USA).

## Results

### General information

Out of the 195 children initially recruited, 14 were excluded due to their inability to cooperate with the measurement of IOP under topical anesthesia before induction. Ultimately, 181 children (59 cases in the NS group, 62 cases in the EL group, and 60 cases in the EH group) were included for analysis (Fig. 1). As shown in Table 1, there were no significant differences in age, gender, ASA classification, BMI, operation time, and respiratory parameters among the three groups (*P* > 0.05).

### Comparison of IOP

**Fig. 2:**
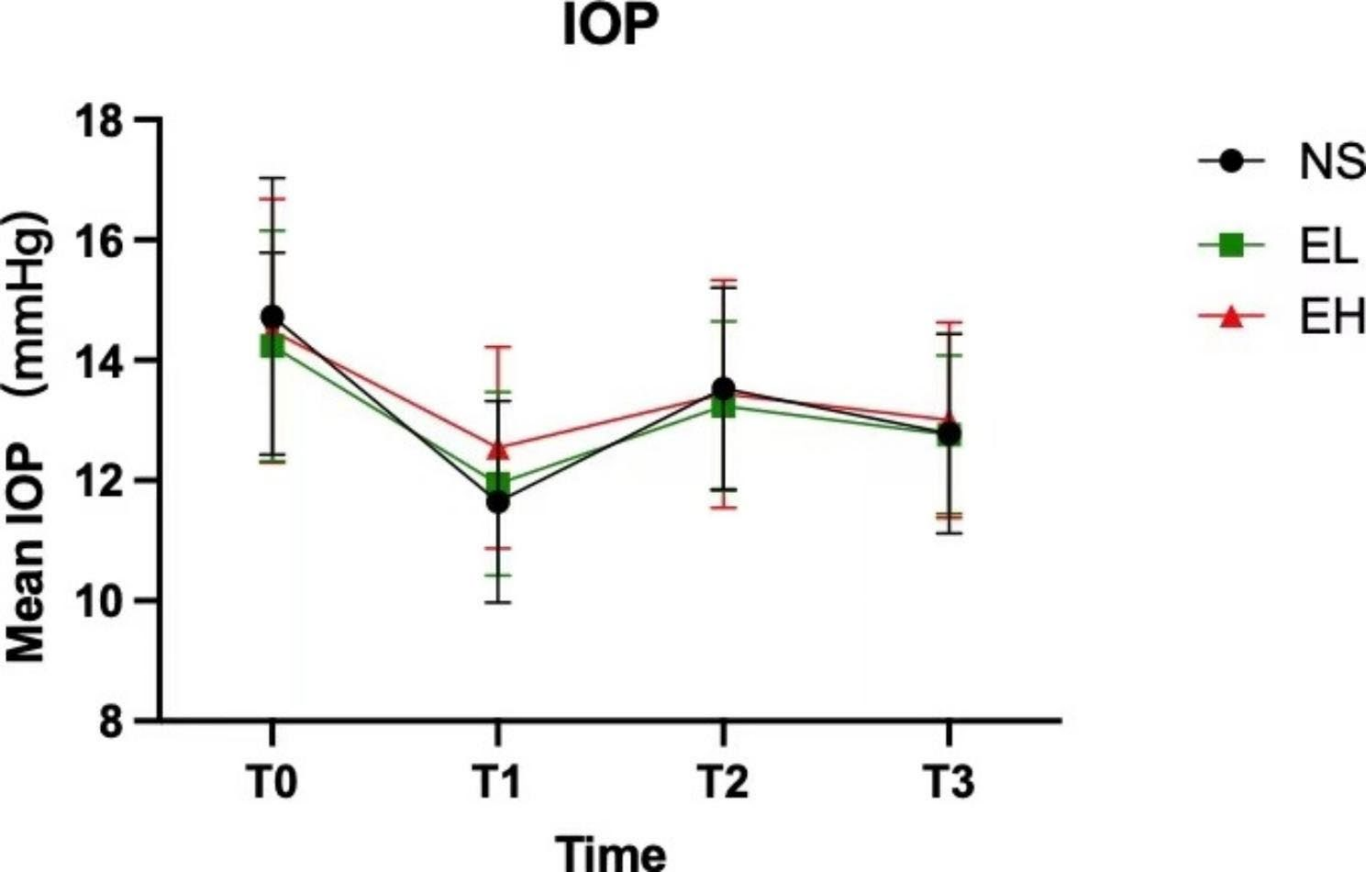
T_0_ = before induction, T_1_ = 1 min after induction but before laryngeal mask insertion, T_2_ = immediately after laryngeal mask insertion, T_3_ = 2 min after laryngeal mask insertion. IOP at T_1_, T_2_ and T_3_ were all lower than that at T_0_. EH group had significantly higher IOP than EL group and NS group at T_1_ (*P* < 0.05)

Figure 2 illustrates that IOP measurements at T_1_, T_2_, and T_3_ were lower than T_0_. Inter-group comparisons at the same time point revealed that the EH group had significantly higher IOP than the EL group and NS group at T_1_, while no such difference was found between the EL group and NS group, nor at other time points (Table 2).

### Comparison of hemodynamic parameters

**Fig. 3:**
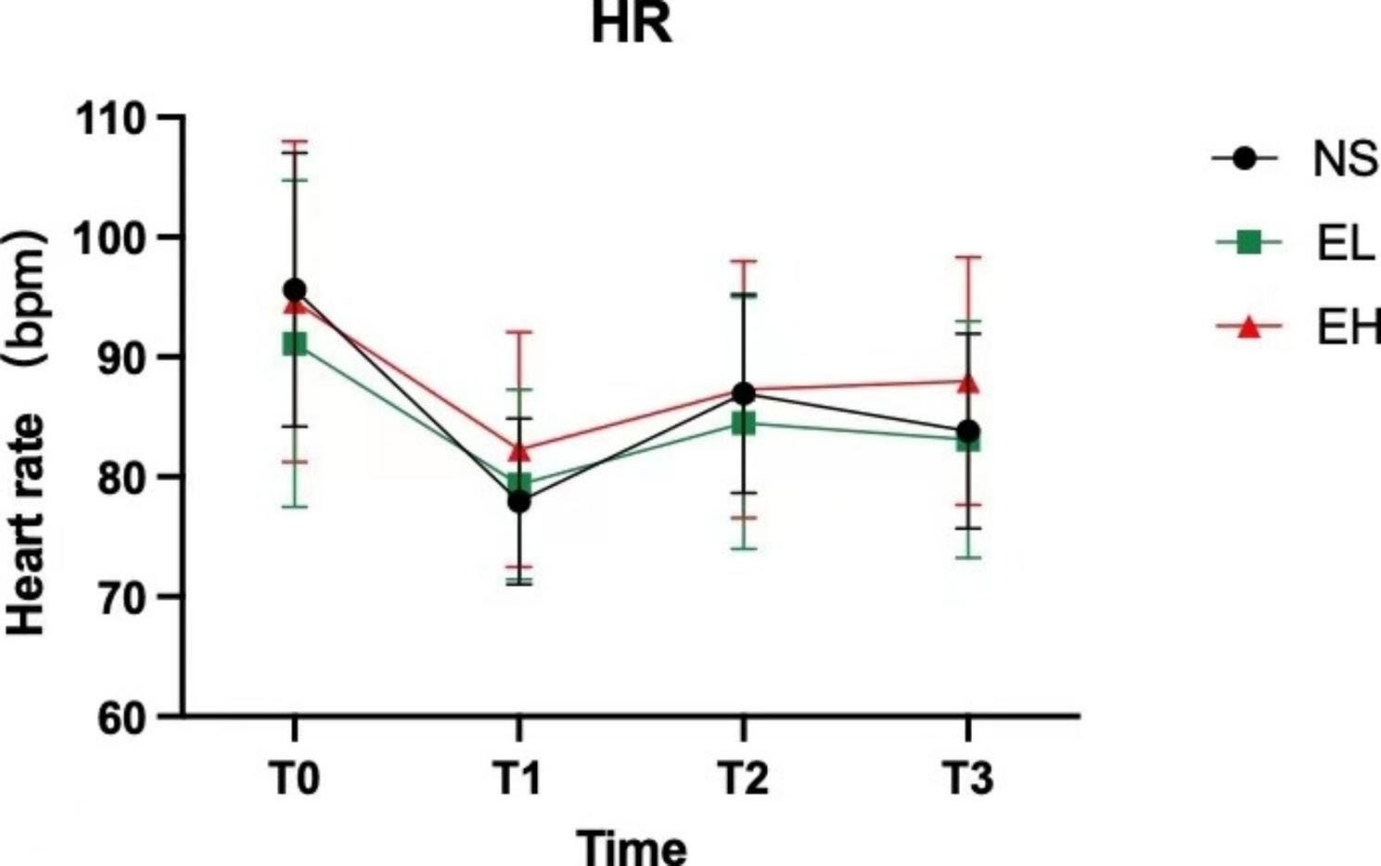
T_0_ = before induction, T_1_ = 1 min after induction but before laryngeal mask insertion, T_2_ = immediately after laryngeal mask insertion, T_3_ = 2 min after laryngeal mask insertion. HR at T_1_, T_2_ and T_3_ were all lower than that at T_0_. EH group had significantly higher HR than EL group and NS group at T_1_ (*P* < 0.05)

HR at T_1_, T_2_, and T_3_ were significantly lower than at T_0_, and HR at T_2_ was higher than at T_1_ (Fig. 3). Furthermore, the EH group had significantly higher HR at T_1_ than the other two groups. No statistical difference was found in DBP among the three groups at any time points.

SBP at T_1_, T_2_, and T_3_ were significantly lower than that at T_0_, and SBP at T_2_ was higher than that at T_1_. No significant differences were found in inter-group comparisons. Additionally, no statistical difference was found in DBP among the three groups at any time points. More details are provided in Table 3.

## Discussion

IOP refers to the pressure exerted by the ocular contents on the walls of the eyeball, typically ranging from 11 to 21 mmHg. Various factors influence IOP, including demographic factors such as age and gender and individual physiological factors such as aqueous humor circulation, extraocular muscle tension, and respiratory circulation [9, 10]. During the induction of general anesthesia, using anesthetic drugs and procedures can induce changes in the patient’s physiological state, ultimately affecting IOP through the production and circulation of aqueous humor [11].

Pediatric ophthalmic surgery often requires general anesthesia, and anesthesiologists must ensure the stability of IOP during the induction period to prevent sudden fluctuations. Commonly used drugs for intravenous induction of anesthesia in children include propofol, opioids, and esketamine. In current clinical settings, the combination of propofol and sufentanil is frequently employed, which has been shown to significantly reduce IOP [12, 13]. The mechanism underlying this effect may involve the γ-aminobutyric acid (GABA) receptor agonism of propofol, which, in conjunction with opioid receptors in the eye, can inhibit ciliary epithelial secretion, decrease the production and discharge of aqueous humor, and relax extraocular muscles, thereby reducing IOP.

The pharmacological mechanism of esketamine, similar to ketamine, involves the stimulation of sympathetic nerves by blocking N-methyl-D-aspartate (NMDA) receptors, leading to increased aqueous humor production and extraocular muscle tension, as well as restricted aqueous humor outflow, ultimately resulting in elevated IOP [14]. Given its potential for increasing IOP, the use of esketamine in pediatric ophthalmic anesthesia has been limited by many anesthesiologists and ophthalmologists.

There are benefits when using esketamine in combination with other anesthetic drugs. Esketamine has a certain sedative and analgesic effect, which can reduce the dosage of other drugs (e.g., opioids, propofol, etc.) in anesthesia induction [15]. Zhan et al.[16] reports that combination of 0.2 mg/kg esketamine and propofol was effective and safe in painless gastrointestinal endoscopy as evidenced by less propofol consumption per minute, shorter induction time, and lower incidence of cough and body movement relative to propofol alone. Furthermore, esketamine can prevent propofol-induced injection pain [17].

Limited studies have investigated the effect of esketamine in combination with other commonly used anesthetics on IOP. Therefore, this study aimed to explore the effect of different doses of esketamine combined with propofol and sufentanil on IOP during the induction of general anesthesia, which appears to be innovative and practical.

The study results revealed that the EH group had significantly higher IOP than the other two groups, indicating that esketamine could elevate IOP. However, the IOP of all three groups after induction was lower than at baseline, suggesting that the IOP-reducing effect of propofol and sufentanil was stronger than the IOP-elevating effect of esketamine. Therefore, it is believed that administering esketamine at a dosage not exceeding 0.5 mg/kg, in combination with propofol and sufentanil, does not elevate IOP compared to the pre-induction status. Additionally, the study revealed that the impact of esketamine on IOP was dose-dependent, consistent with the findings of Ausinsch’s study [18].

Using a laryngeal mask airway (LMA) during induction in this study helped reduce the elevation of IOP caused by tracheal intubation in ophthalmic surgery [19]. There were no significant differences in IOP between T_2_ (immediately after laryngeal mask insertion) and T_3_ (2 min after laryngeal mask insertion) in all three groups, and the IOP values were lower than those before induction. This finding indicates that the three-drug combination could inhibit the elevation of IOP caused by laryngeal mask insertion. Furthermore, it was observed that IOP increased at T_2_ in each group and then decreased, suggesting that although the LMA insertion might cause some irritation, it was not sufficient to reverse the IOP-reducing effect of the induction drugs.

Prior studies have identified BP as one of the important factors of IOP [20], but no relationship between heart rate and IOP has been found. The results of this study demonstrated that HR and IOP at T_1_ in the EH group were higher than in the other two groups, while no significant differences were found in BP. Based on these results, it can be inferred that HR plays a major role in increasing cardiac output in children, suggesting that the change in IOP is associated with cardiac output rather than BP alone. Furthermore, the wall of the ophthalmic vein is thinner than that of the ophthalmic artery and is more likely to expand and transmit increased pressure [21]. Consequently, compared to systolic blood pressure, IOP is more vulnerable to changes in venous return, and increased cardiac output can raise venous pressure, thereby affecting aqueous humor outflow. In contrast with the literature [22], no effect of esketamine on increasing BP was found in this study, which might be attributed to the different cardiovascular effects of esketamine and ketamine. Additionally, since the subjects of this study were children with high sympathetic excitability, the drug had little effect on BP.

This study made several improvements based on previous research. Sobczak et al. [23] revealed an association between IOP and corneal thickness, demonstrating that children under 6 years of age had significantly lower IOP than older children due to the lower hardness of the cornea. In this study, pediatric patients aged 7 to 12 years were included, as IOP tends to be more consistent within this age range. Some researchers have suggested that the increase in IOP might be related to the hypercapnia induced by ketamine [24]. To mitigate the influence of CO_2_ on IOP, PETCO_2_ was maintained at a consistent level by assisted ventilation in our study.

Some limitations present in this study should be acknowledged. Firstly, we did not use bispectral index (BIS) monitoring to assess the depth of anesthesia, as the use of multiple drugs, especially esketamine, may affect BIS readings [25, 26]. Secondly, using esketamine might result in various degrees of analgesia in different groups. Thirdly, invasive arterial pressure monitoring was not performed in this study, which may have hindered the real-time assessment of circulatory status.

## Conclusion and prospectives

The induction of general anesthesia in children undergoing pediatric strabismus surgery using propofol and sufentanil, along with a maximum dose of 0.5 mg/kg esketamine, does not result in an elevation of IOP compared to the pre-anesthesia status. However, further studies are needed to determine the efficacy and suitability of using esketamine in children with other eye conditions, such as glaucoma. In conclusion, anesthesiologists should choose the optimal combination of anesthetics based on individual clinical conditions and surgical requirements.

**Table 1:** Comparison of general information among the three groups

	NS group (n = 59)	EL group (n = 62)	EH group (n = 60)
Age (yrs)	8.5 ± 1.6	8.7 ± 1.8	8.8 ± 1.7
Gender (M/F)	33/26	27/35	26/34
ASA grade (I/II)	45/14	52/10	48/12
BMI (kg/m^2^)	19.5 ± 4.5	18.2 ± 3.7	18.3 ± 5.5
Operation time (min)	18.7 ± 7.2	17.3 ± 5.5	17.1 ± 6.4
VT (ML)	191.8 ± 59.4	191.5 ± 57.1	181.6 ± 62.5
RR (beat/min)	19.5 ± 4.1	18.6 ± 3.6	18.3 ± 2.9
PETCO_2_ (mmHg)	41(35–45)	40(35–45)	40(35–45)

Note: Data were presented as mean ± SD or counts. ASA = American society of anesthesiologists, BMI = body mass index, VT = tidal volume, RR: respiratory rate, PETCO_2_ = pressure of end-tidal CO_2_.

**Table 2:** Comparison of IOP among the three groups

		T_0_	T_1_	T_2_	T_3_	F	*P*
IOP	NS group	14.7 ± 2.3	11.6 ± 1.7^a^	13.5 ± 1.7^ab^	12.8 ± 1.7^abc^	208.800	0.000
(mmHg)	EL group EH group F *P*-Value	14.3 ± 1.9 14.5 ± 2.1 0.741 0.478	12.0 ± 1.6^a^ 12.6 ± 1.6^a#^ 5.261 0.006	13.2 ± 1.5^ab^ 13.4 ± 1.8^ab^ 0.4082 0.666	12.8 ± 1.4^abc^ 13.0 ± 1.6^abc^ 0.491 0.613	120.100 76.160	0.000 0.000

Note: Data were presented as mean ± SD. T_0_ = before induction, T_1_ = 1 min after induction but before laryngeal mask insertion, T_2_ = immediately after laryngeal mask insertion, T_3_ = 2 min after laryngeal mask insertion

# indicated *P* value less than 0.05 compared with NS group

Overall test, inter-group (F, *P*) 0.619, 0.540, *P* > 0.05; time (F, *P*) 293.848, 0.000, *P* < 0.05; interaction (F, *P*) 6.723, 0.000, *P* < 0.05

**Table 3:** Comparison of hemodynamic parameters among the three groups

				T_2_	T_3_	F	*P*
HR	NS group	95.6 ± 11.4	77.9 ± 6.9^a^	86.9 ± 8.3^ab^	83.8 ± 8.2^abc^	118.000	0.000
(beat/min)	EL group	91.6 ± 13.6	79.8 ± 7.9^a^	84.9 ± 10.6^ab^	83.9 ± 10.4^ab^	54.063	0.000
	EH group	94.2 ± 13.4	81.8 ± 9.8^#a^	86.6 ± 11.0^ab^	87.1 ± 10.2^ab^	58.439	0.000
	F	1.479	3.25	0.673	2.153		
	*P*	0.231	0.041	0.512	0.119		
SBP (mmHg)	NS group	105.9 ± 11.6	91.2 ± 7.7^a^	98.2 ± 7.3^ab^	92.8 ± 5.1^ac^	34.673	0.000
	EL group	103.7 ± 8.9	91.5 ± 9.4^a^	99.9 ± 6.9^ab^	94.0 ± 6.9^ac^	28.788	0.000
	EH group	106.5 ± 11.5	93.2 ± 8.3^a^	98.5 ± 8.0^ab^	93.6 ± 6.3^ac^	30.694	0.000
	F	1.140	0.971	0.906	0.577		
	*P*	0.322	0.381	0.406	0.563		
DBP (mmHg)	NS group	66.2 ± 5.2	63.9 ± 4.3	65.6 ± 5.4	63.7 ± 5.5^a^	3.910	0.010
	EL group	64.7 ± 5.7	64.9 ± 4.9	63.3 ± 4.4	64.4 ± 4.5	1.515	0.212
	EH group	65.5 ± 4.5	65 ± 3.8	64.5 ± 5.6	64.9 ± 5.0	0.527	0.665
	F	1.281	1.055	2.924	0.795		
	*P*	0.28	0.35	0.56	0.453		

Note: Data were presented as mean ± SD. T_0_ = before induction, T_1_ = 1 min after induction but before laryngeal mask insertion, T_2_ = immediately after laryngeal mask insertion, T_3_ = 2 min after laryngeal mask insertion

Overall test for HR, inter-group (F, *P*) 0.914, 0.540, *P* > 0.05; time (F, *P*) 219.4, 0.000, *P* < 0.05; interaction (F, *P*) 5.98, 0.000, *P* < 0.05

Overall test for SBP, inter-group (F, *P*) 0.503, 0.606, *P* > 0.05; time (F, *P*) 92.168, 0.000, *P* < 0.05; interaction (F, *P*) 1.069, 0.194, *P* > 0.05

Overall test for DBP, inter-group (F, *P*) 0.938, 0.393, *P* > 0.05; time (F, *P*) 2.061, 0.106, *P* > 0.05; interaction (F, *P*) 1.797, 0.098, *P* > 0.05

a indicated *P* value less than 0.05 compared with T_0_, b indicated *P* value less than 0.05 compared with T_1_, c indicated *P* value less than 0.05 compared with T_2_

# indicated *P* value less than 0.05 compared with NS group, ## indicated *P* value less than 0.05 compared with EL group

## Data Availability

The datasets used and analyzed in the current study are available from the corresponding author on reasonable request.
